# Influence of Implantation Depth on the Performance of Intracortical Probe Recording Sites

**DOI:** 10.3390/mi12101158

**Published:** 2021-09-27

**Authors:** Joshua O. Usoro, Komal Dogra, Justin R. Abbott, Rahul Radhakrishna, Stuart F. Cogan, Joseph J. Pancrazio, Sourav S. Patnaik

**Affiliations:** Department of Bioengineering, The University of Texas at Dallas, Richardson, TX 75080, USA; joshua.usoro@utdallas.edu (J.O.U.); komal.dogra@utdallas.edu (K.D.); justin.abbott@utdallas.edu (J.R.A.); radhakrishnar@utdallas.edu (R.R.); sxc149830@utdallas.edu (S.F.C.); Sourav.Patnaik@UTDallas.edu (S.S.P.)

**Keywords:** microelectrode array, single unit yield, single shank, multi-shank, depth-dependence

## Abstract

Microelectrode arrays (MEAs) enable the recording of electrical activity from cortical neurons which has implications for basic neuroscience and neuroprosthetic applications. The design space for MEA technology is extremely wide where devices may vary with respect to the number of monolithic shanks as well as placement of microelectrode sites. In the present study, we examine the differences in recording ability between two different MEA configurations: single shank (SS) and multi-shank (MS), both of which consist of 16 recording sites implanted in the rat motor cortex. We observed a significant difference in the proportion of active microelectrode sites over the 8-week indwelling period, in which SS devices exhibited a consistent ability to record activity, in contrast to the MS arrays which showed a marked decrease in activity within 2 weeks post-implantation. Furthermore, this difference was revealed to be dependent on the depth at which the microelectrode sites were located and may be mediated by anatomical heterogeneity, as well as the distribution of inhibitory neurons within the cortical layers. Our results indicate that the implantation depth of microelectrodes within the cortex needs to be considered relative to the chronic performance characterization.

## 1. Introduction

Intracortical microelectrode arrays (MEAs) are capable of recording the electrical activity of neurons within the cortex with high temporal and spatial resolution for single unit activity (SUA) [1], thus enabling a variety of brain-machine interface applications [2,3,4,5,6,7]. Advances in micro-scale manufacturing have enabled a wide design space for these devices relative to materials, size, and complexity [8,9,10,11]. In general, probes fall into one of two configurations: single shank (SS) or multi-shank (MS). While a SS probe is capable of stimulating and recording at various depths throughout the cortex, the MS MEAs aim to spatially distribute the recording sites normal to the insertion plane. By increasing the number of shanks on the neural microelectrodes however, there is a marked increase in device volume and altered mechanical coupling to the brain. Szarowski et al. performed a comprehensive analysis of brain histological responses to devices with varied MEA shank cross-sectional areas of 16,900, 10,000, and 5000 μm^2^ (among other physical properties) and reported that the initial tissue response is proportional to the device size, whereas the sustained response is most likely a result of tissue-material interactions [12]. Wang et al. reported that buckling forces for a MS array did not increase proportionally with an increased number of shanks [13], however the shear forces, quantified during implant insertion in cortical tissue, increased linearly with a larger number of shanks. This increase in shear forces, as demonstrated by a recent in vitro study [14], may lead to an increase in astrocytic cell density and decrease in neurite viability which could have implications on the local immunological environment surrounding indwelling probes. Numerous studies have demonstrated that these contributions to neuronal injury and gliosis in vivo are also heightened in the presence of larger probes as well [12,15,16], exacerbating the inflammatory cascade that may lead to a decrease in device performance. While the histological response to SS and MS MEAs has been well-studied, few studies have directly compared the device performance outcomes between the two configurations. In this brief report, we aim to quantify the differences in the electrophysiological and electrochemical characteristics of SS and MS neural probes implanted in the rat motor cortex. Our results indicate that SS and MS arrays exhibit a differential decay profile relative to the proportion of the active microelectrodes, an effect that appears to be related to the microelectrode site depth within the cortex rather than the number of shanks. These findings have implications for understanding how different microelectrode array geometries perform under chronic implantation conditions.

## 2. Materials and Methods

### 2.1. Devices

**Figure 1:**
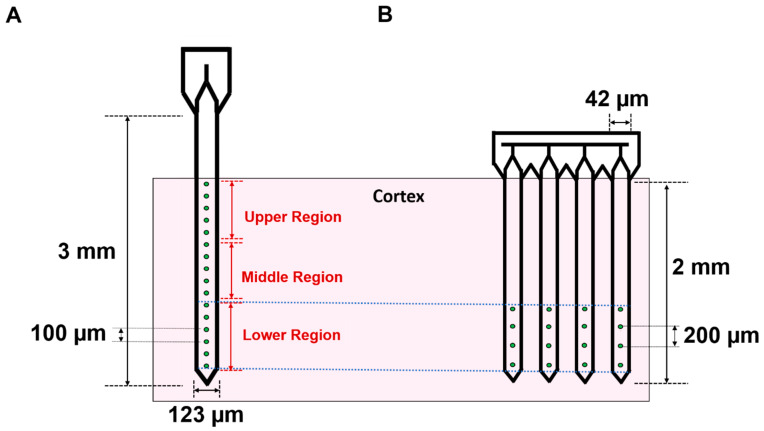
Dimensions and configurations for single shank and multi-shank microelectrode arrays. Depth-based differences in microelectrode placement for single shank (**A**) and multi-shank (**B**) microelectrode devices, highlighting the differences between experimental groups. Pink shaded region indicates the cortex of the brain while dashed lines indicate that microelectrodes in the lower third of single shank arrays are implanted and aligned to the depth of multi-shank microelectrodes.

Experiments were carried out using commercially available silicon devices (Neuronexus Technologies, Ann Arbor, MI, USA). SS arrays (A1x16-3 mm-100-177-CM16LP) were 3 mm in length, 15 µm thick, and a maximum of 123 µm wide at the base of the shank (Figure 1A). These devices have 16 iridium microelectrodes with a geometric surface area of 177 µm^2^ and spaced 100 µm apart, spanning approximately 1.5 mm from the tip of the shank. MS arrays (A4x4-2 mm-200-200-200-CM16LP) were comprised of 4 shanks, each 2 mm in length, 15 µm thick, and a maximum of 42 µm wide at the base of the shank (Figure 1B). Each shank contained 4 iridium microelectrodes that were 200 µm^2^ and spaced 200 µm apart, spanning approximately 0.6 mm from the tip of the shank. MS microelectrode sites were activated prior to implantation using a previously established process consisting of rectangular potential pulsing between −0.6 V and 0.8 V versus Ag|AgCl in PBS pH 7.4 [17] to reduce impedance, thereby helping to ensure a comparable performance of the novel custom-designed arrays.

### 2.2. Surgical Implantation of Devices

**Figure 2:**
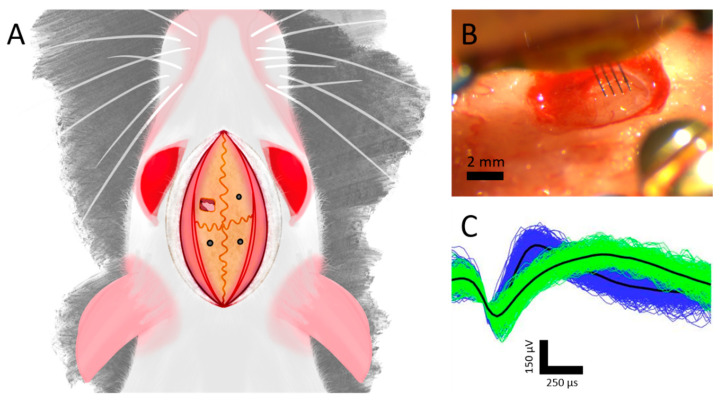
Neuronal data acquisition and analysis. (**A**) Implantation schematic with a craniotomy over the left motor cortex and bone screws in the adjacent quadrants. (**B**) Representative image during a multi-shank implantation. (**C**) Representative single units recorded simultaneously from the same microelectrode of a single shank device.

All animal procedures were approved by The University of Texas at Dallas Institutional Animal Care and Use Committee. Adult, female Sprague Dawley rats (Charles River Wilmington, Laboratories, Inc., Wilmington, MA, USA) were implanted with either a SS (*n* = 5) or MS (*n* = 5) array. Animals were initially anesthetized using an intraperitoneal injection of a ketamine (65 mg/kg), xylazine (13.33 mg/kg), and acepromazine (1.5 mg/kg) cocktail (KXA, 0.94 mL/kg), followed by an intramuscular injection of atropine sulfate (0.093 mL/kg) (Med-Vet International, Mettawa, IL, USA). After deep anesthesia was achieved, confirmed by tail and toe pinches, the rats’ scalp was shaved to eliminate hair from the surgical site. Rats were then transferred to a stereotactic frame (Kopf Instruments, Tujunga, CA, USA) where anesthesia was maintained using a maximum of 2% isoflurane (Vedco Inc., St Joseph, MO, USA) mixed with 100% oxygen. Alternating rounds of 10% iodine and 70% ethanol were used to sterilize the scalp and ophthalmic ointment (Lubrifresh P.M., Medline, Northfield, IL, USA) was placed over the eyes to prevent drying and irritation. After the initial midline incision and resecting of tissue, 3 anchoring bone screws (Stoelting Co., Wood Dale, IL, USA) were placed in the quadrants (defined by bregma and the coronal and sagittal sutures) adjacent to site of implantation as seen in Figure 2A. A ~2 mm × 2 mm craniotomy was created in the left motor cortex centered above the forepaw representation within the cortex (~2 mm anterior from bregma and ~2 mm lateral from the midline) (Figure 2A). After the dura was resected, stainless steel ground and reference wires from the array were wrapped around the bone screws, and the device was inserted to a depth of 1.5–1.7 mm using an electronically controlled micropositioner (Kopf Instruments, Actuated Medical, Bellefonte, PA, USA). Collagen-based dural grafts (Biodesign^®^ Dural Graft, Cook Medical LLC., Bloomington, IN, USA) were placed around the device to serve as a dura replacement, and the craniotomy was sealed in a topical adhesive. Dental cement (Stoelting Co., Wood Dale, IL, USA) was then applied on the skull to form a head cap that encapsulated the device and bone screws, and surgical staples were used to close the initial midline incision. After surgery, rats were injected with 0.15 mL/kg of buprenorphine (ZooPharm, LLC., Windsor, CO, USA), 0.05 mL/kg of cefazolin (Med-Vet International, Mettawa, IL, USA), and ~3 mL of sterilized phosphate-buffered saline (PBS) to aid with rehydration. Rats were given a follow-up injection of buprenorphine 72 h after surgery.

### 2.3. Electrophysiological Recordings and Analysis

Weekly electrophysiological recordings were conducted on anesthetized animals starting one-week post-surgery for 8 weeks per previously established protocol [18]. Wideband recordings of spontaneous activity within the motor cortex were recorded simultaneously from all 16 microelectrodes for 10 min at 40 kHz (Omniplex, Plexon, Inc., Dallas, TX, USA). Data were then processed using a 4-pole, Butterworth high pass filter with a cutoff frequency of 250 Hz to eliminate local field potential contributions. Individual spike waveforms were then extracted using a −4σ threshold from the root mean square (RMS) of the filtered wide band signal. Single units were then discriminated based on separation in principal component space, and whether the collected waveforms contained at least 100 spikes with a <3% violation of a 1.5 ms minimum refractory period. Only putative units with V_pp_ greater than 40 µV were included for further analysis. The signal-to-noise ratio (SNR) was calculated by dividing the peak-to-peak voltage (V_pp_) of each unit by the RMS noise of the corresponding channel [18]. Microelectrodes on SS devices were also grouped into upper, middle, and lower thirds to explore potential depth-dependent effects on recording, and to provide a more direct comparison to the MS arrays, which have microelectrodes concentrated in the deeper regions of the cortex upon implantation (Figure 1). 

### 2.4. Electrochemical Measurements and Analysis

Electrochemical impedance spectroscopy (EIS) measurements were performed in anesthetized animals immediately following electrophysiological recordings using either a Gamry Reference 600 Potentiostat (Gamry Instruments, Warminster, PA, USA) or CH Instruments 604e series Electrochemical Analyzer/Workstation (CH Instruments Inc., Austin, TX, USA). EIS was performed using a 10 mV RMS sinusoidal signal versus external Pt counter wire and Ag|AgCl reference electrodes. Signals were acquired over a range of 1 to 10^5^ Hz at 10 points per decade. A custom MATLAB script (Mathworks, Natick, MA, USA) was utilized to extract the impedance magnitude (|Z|) at 1 kHz [19]. 

### 2.5. Immunohistochemistry

Immunohistochemistry (IHC) preparation was performed as previously described. Briefly, animals were euthanized using 200 mg/kg IP injection of sodium pentobarbital. Toe and tail pinches were used to confirm unconsciousness, and a transcardial perfusion was performed with PBS and followed by 4% paraformaldehyde (PFA) (Sigma-Aldrich, St. Louis, MO, USA). Intact brains were extracted and submerged in PFA solution for at least 24 h prior to processing. Afterwards, the brain was sectioned around the implantation site and placed in a 4% agarose solution (m/V) (Sigma-Aldrich, St. Louis, MO, USA) for better handling. Axial slicing of the sectioned tissue was performed using a vibratome (VT 1000S, Leica vibratome, Wetzlar, Germany) and 100 µm thick slices were collected using paint brushes. Tissue slices were stored in PBS with 0.1% (*w*/*v*) sodium azide (Sigma-Aldrich, St. Louis, MO, USA) at 4 °C until staining was performed.

Brain slices were blocked in 4% (*v*/*v*) normal goat serum (Abcam Inc., Cambridge, UK) with 0.3% (*v*/*v*) Triton X-100 in 1× PBS with 0.1% sodium azide (Sigma-Aldrich, St. Louis, MO, USA) for one hour. Afterwards, slices were incubated overnight at 4 °C with primary antibody solutions (buffered solution with 3% (*v*/*v*) Triton X-100 in 1× PBS) that target astrocytes (glial fibrillary acidic protein (GFAP)) and neuronal nuclei (NeuN) (Abcam Inc., Cambridge, UK) as detailed in Table 1. The next day, slices were washed and then incubated with blocking solution containing goat anti-chicken IgY (Alexa Fluor 647), goat anti-rabbit IgG (Alexa Fluor 555) (1:4000 dilution) and DAPI (0.6 µM) (Abcam Inc., Cambridge, UK). Slices were then washed and mounted on glass slides with Fluoromount aqueous mounting medium (Sigma-Aldrich, St. Louis, MO, USA). 

Stained slices were viewed under an inverted confocal microscope (Nikon Ti eclipse + A1R, Nikon Instruments Inc., Tokyo, Japan) and controlled by Nikon Instruments Software package (version AR 4.40.00). Images were collected at 2048 × 2048 transverse resolution using a 10× objective. All hardware and software settings were conserved across individual image acquisitions. 

### 2.6. Statistical Analysis

Statistical analysis was performed in OriginPro 2021 (Origin Lab, Northampton, MA, USA) and MATLAB R2020a (MathWorks, Natick, MA, USA). Unless otherwise noted, all statistics were expressed as mean ± standard error of the mean. A test of proportions z-test was utilized to compare the proportion of active microelectrodes between single and multi-shank groups, and across depth-based groups (upper, middle, and lower), respectively. When applicable, data were binned in two-week intervals and averaged across microelectrodes. Mann–Whitney U tests were used to compare SS vs. MS data at individual time points whereas the Kruskal–Wallis (nonparametric ANOVA) test was used to explore depth-related differences between groups. Dunn’s tests were used as a follow up to determine differences between groups analyzed in the Kruskal–Wallis ANOVA. For all conditions, a *p*-value <0.05 was considered statistically significant. 

## 3. Results

**Figure 3:**
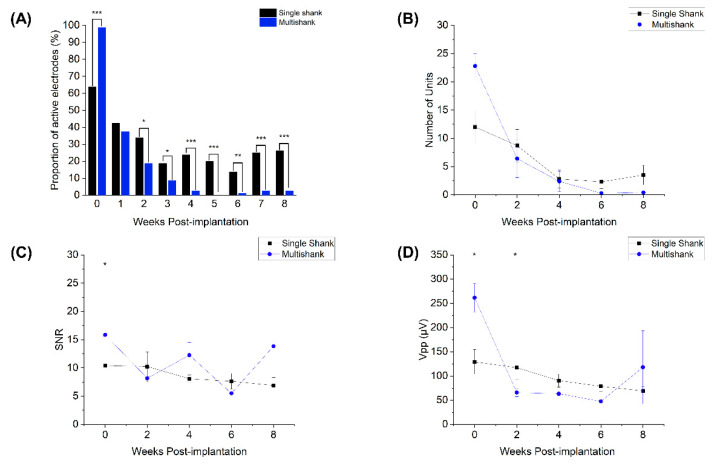
Intracortical microelectrode array performance. The proportion of active microelectrodes (**A**), number of units (**B**), signal-to-noise ratio (**C**) and peak-to-peak voltage (**D**) of single shank (black) and multi-shank (blue) arrays. Data for active microelectrodes from both device configurations were represented as a proportion (test of proportions *z*-test) and the rest of the data was presented as mean ± SEM (Mann–Whitney U tests). * indicates *p* < 0.05, ** indicates *p* < 0.01, and *** indicates *p* < 0.001.

To evaluate the differences in recording capabilities between single and multi-shank devices, electrophysiological data were recorded from rats implanted with either SS (*n* = 5) or MS (*n* = 5) Neuronexus arrays. We observed that immediately following implantation, approximately 64% of microelectrodes on SS arrays were able to record single unit activity, whereas nearly all the microelectrodes on MS arrays exhibited activity (Figure 3A; 64% vs. 99%; test of proportions z-test, *p* < 0.05). Similarly, MS arrays recorded nearly twice as many single units from presumptive individual neurons as SS arrays immediately after implantation (Figure 3B; 22.8 ± 2.2 vs. 12.0 ± 2.9, *p* < 0.05) and yielded a higher SNR as well (Figure 3C). V_pp_ for MS arrays was higher in the initial week, but dropped by almost four times during the subsequent recording periods (261 ± 29 µV at week 0 vs. 68 ± 9 µV at week 2; *p* < 0.05); whereas the V_pp_ for SS arrays remained consistent throughout the study period (Figure 3D). Within 4 weeks after surgery, however, MS array recording performance rapidly declined, whereas SS array performance remained relatively stable. The proportion of active microelectrode sites for SS arraysremained significantly higher over the last 4 weeks of the study. While SNR for both types of devices did decrease slightly over time, there were few differences seen between the two arrays, indicating that the diminished recorded activity for MS arrays may stem from a lack of nearby active neurons, rather than an inability to resolve the unit activity. 

**Figure 4:**
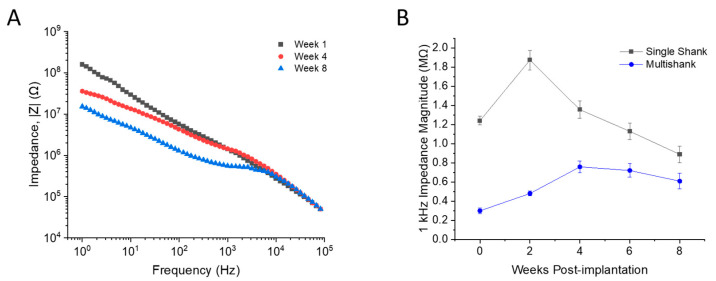
Electrochemical impedance spectroscopy. (**A**) Impedance magnitude for a representative single shank microelectrode at Weeks 1, 4, and 8. (**B**) Impedance magnitude at 1 kHz for single shank (black) and multi-shank (blue) devices. Data presented as mean ± SEM.

The electrochemical stability of the devices was evaluated using electrochemical impedance spectroscopy, wherein the impedance magnitude of all microelectrodes across all devices was measured weekly. At the 1 kHz frequency, impedance magnitudes were between 0.4–2 MΩ (Figure 4B), well within the range of values often associated with the ability for devices to resolve and record SUA. SS microelectrode sites exhibited an increase in mean impedance at 1 kHz from in vitro (1.24 ± 0.05 MΩ) to 1 week post-implantation (1.90 ± 0.67 MΩ) which is consistent with prior observations comparing in vitro and in vivo impedances [17,20]. Overall, our results are consistent with previous literature investigating the chronic longevity of Neuronexus probes [21,22].

Lastly, we investigated potential depth-related differences in electrophysiological recordings. SS microelectrodes were divided into upper, middle, and lower thirds based on their depth upon implantation within the cortex. Because both types of MEAs were implanted to a depth of ~1.5–2 mm, the lower third of SS microelectrodes corresponded to the depth at which MS microelectrodes were implanted, approximately situated in layers V/VI of the cortex [23,24,25,26]. Middle third microelectrodes were located roughly in layers III/IV while the upper third of the microelectrodes were located in layer II–III. 

**Figure 5:**
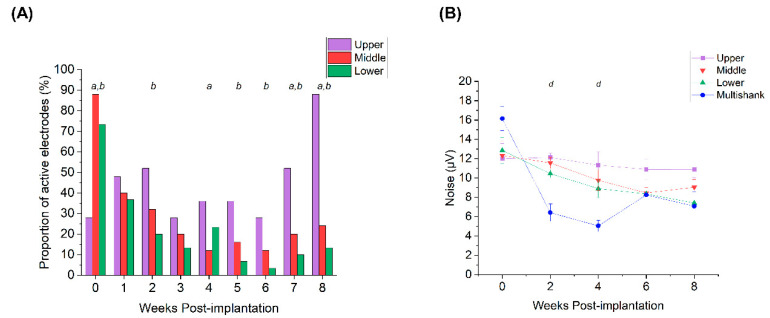
Depth-dependent electrophysiological measures. (**A**) Proportion of active microelectrodes for upper (violet), middle (red), and lower (green) microelectrodes located in the SS arrays. The annotation *a* denotes significant differences between upper and middle groups, *b* denotes significant differences between upper and lower groups, and *c* denotes significant differences between middle and lower groups, respectively (test of proportions *z*-test, *p* < 0.05). (**B**) Noise levels for the microelectrodes at different depths. The annotation *d* indicates significant differences during Week 2 and Week 4 between upper third and multi-shank microelectrodes. Data presented as the mean ± SEM.

Figure 5A shows that there was a significant difference (test of proportions *z*-test, *p* < 0.05) between groups at all time points, except for Week 1 and Week 3. Immediately following implantation (Week 0), the proportion of microelectrodes exhibiting single unit activity was higher in the middle group, followed by the lower and upper groups, respectively (88% vs. 73% vs. 28%; test of proportions z-test, *p* < 0.05). Lower and middle microelectrode groups exhibited higher single unit activity in the initial weeks, but gradually declined during the 8-week period. Towards the end of the study duration, upper third microelectrodes showed significantly greater recorded activity than the middle and lower third microelectrodes. It is important to note the virtually identical decay profiles between the active MS microelectrode sites compared to the lower group of SS microelectrodes. This observation suggests a depth-related phenomenon may underly the observed overall differences between proportion of active microelectrodes from SS and MS arrays. Additionally, we did not observe notable differences in V_pp_ at varying depths, other than Week 0 (261 ± 29 µV for MS arrays vs. 106 ± 19 µV for upper group vs. 125 ± 21 µV for middle group vs. 138 ± 36 µV for lower group; *p* < 0.05). Noise levels, however, were significantly higher for the upper third microelectrodes compared to the MS microelectrodes at Week 2 (12.1 ± 0.4 µV vs. 6.4 ± 0.9 µV; *p* < 0.05) and Week 4 (11.3 ± 1.3 µV vs. 5.1 ± 0.6 µV; *p* < 0.05) (Figure 5B), respectively, indicating potential shifts in noise after the resolution of the acute phase of the foreign body response. Despite the differences in iridium activation between MS and SS microelectrodes, the noise level for MS microelectrodes was similar to the lower and middle third of microelectrodes on SS devices.

**Figure 6:**
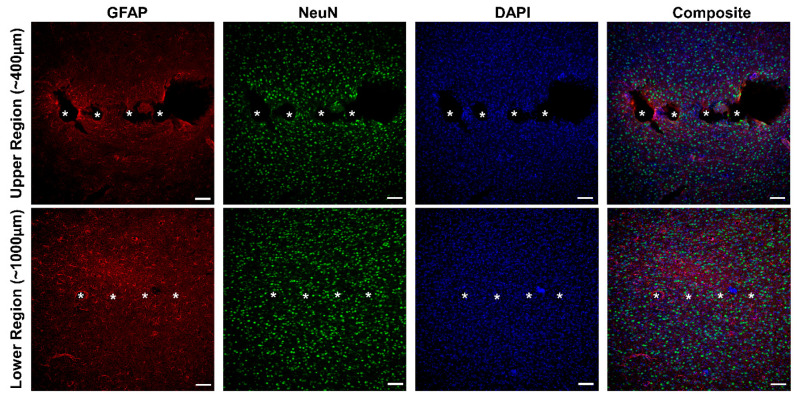
Immunohistological profile of cortical tissue implanted with multi-shank arrays at two depths. The columns indicate activated astrocytes/macrophages (GFAP), neuronal nuclei (NeuN), cell bodies (DAPI) and Composite images. Rows represent tissue collected at superficial (top panel, ~400 µm) and deeper (bottom panel, ~1000 µm) depths. The white asterisks (*) on the individual images in the panel indicate the presumptive sites of the device shanks. Scale bar = 100 µm.

Since the tissue response has long been considered a factor in chronic device performance, we performed preliminary IHC focusing on the astrocytic (GFAP) and neuronal nuclei (NeuN) profiles to assess whether or not deeper regions would exhibit more profound tissue damage. For MS arrays, preliminary observations seen in Figure 6 indicate that the astrocytic response was more prominent at superficial depths from the cortical surface (~400 µm) as compared to the deeper regions (~1000 µm). 

Likewise, the neuronal distribution was slightly more pronounced at superficial depths as well. While these findings are preliminary, our observations are consistent with previous studies that examined the foreign body response of silicon-based probes [22,27,28], where superficial depths tend to show a more pronounced immunohistological response than deeper regions. Since the MS microelectrode sites are located within deeper regions in the cortex when implanted, the loss of activity may not be entirely related to a local tissue response.

## 4. Discussion

Efforts in recent years have aimed to improve the functionality and chronic performance of intracortical MEAs through a variety of design considerations. Advances in materials science have enabled devices comprised of soft or softening material [29,30,31,32] or that are coated with biomimetic gels and neuroprotective agents [28,33,34]. Likewise, innovations in fabrication techniques have facilitated the development of ultrasmall arrays [15,35,36], as well as arrays with increased microelectrode density [37,38,39] and unique mechanical/actuation properties. While the effects of these novel design changes have been at the forefront of investigation for intracortical MEAs, the influence of location of microelectrode sites has rarely been considered relative to performance. We observed that while SUA recorded on SS probes remained relatively stable over the 8-week study duration, activity recorded from MS probes rapidly declined over time. Upon further analysis, we observed that SS microelectrodes in the lower third of the shank, implanted to a similar depth as MS probes, exhibited this same severe drop off in activity (Figure 5A). Our results are in close agreement with observations seen by Golabchi et al., which revealed a similar depth dependence in active recording sites in the visual cortex [28]. Interestingly, in that study, coating SS probes with the neuroadhesive protein L1 increased neuronal and axonal density near the implant while reducing glial activation and effectively eliminated the prominent decay of recording activity at the deeper implantation region [28]. Our histological results however (Figure 6), suggest that the foreign body response, characterized by increased concentrations of activated astrocytes, was less pronounced at deeper depths and revealed the presence of neurons in proximity to the shank. While this observation has been commonly shown in the literature, given that the geometric dimensions of the shank taper towards the tip, it does suggest that there may be additional mechanisms involved in the depth-dependent decrease in recorded activity.

One such pathway may be related to the anatomical inhomogeneity in the cortex. Studies examining the cellular makeup and organization of the cortex have shown that more superficial layers (II–III) contain a slightly greater density of neurons, specifically large-bodied pyramidal neurons, as compared to deeper layers (V/VI) which contain neurons with a larger number of dendritic extensions [23,26,40]. While our histology images do reflect this trend (Figure 6, NeuN), they do not fully explain the decay in recorded activity. Immediately following implantation, MS arrays significantly outperformed SS arrays in terms of recording metrics (Figure 3). Furthermore, in this time frame, microelectrodes located in the lower and middle third of the SS recorded more activity than microelectrodes situated in the more superficial layers, indicating that the density of neurons within cortical layers may not entirely correspond to the level of electrophysiological activity that is being recorded. 

It is possible that the relative proportion of excitatory to inhibitory neurons may play a role in the differences in recorded activity. Beaulieu [23] and Meyer et al. [26] demonstrated that while the distribution of GABAergic (inhibitory) neurons was inhomogeneous between layers, there was a concentration of inhibitory neurons in the upper third of layer II–III and in layer V/VIA. Consequently, microelectrodes located in these zones of inhibition may have experienced a substantial suppression in the recorded neuronal activity. Additionally, this GABA-mediated inhibition, particularly in the deeper layers, may be partially explained by the neuronal response to mechanical perturbation. Literature investigating the cortical mechanotransduction involved in traumatic brain injury has shown that a mechanical or stretch injury substantially increased GABA concentrations in vitro, thereby reducing spontaneous single unit activity [41,42,43]. While intracortical MEA implantation may be better modeled as a stab injury, micromotion-induced tissue strain may produce similar mechanical responses. Because both the SS and MS arrays are fixed at the skull, individual shanks can therefore be modeled as cantilever beams where the floating tips (deeper microelectrodes) would be subject to greater displacement forces [44]. This effect would be differentially observed along the length of the shank due to the tapering shank geometry, which would potentially translate to a higher, and more consistent, inhibitory response at deeper microelectrode sites. Furthermore, Magou et al. demonstrated in vitro that non-injured neurons immediately adjacent to stretch-injured neurons displayed acute hyperexcitability following injury induction, which may help to explain the significantly higher proportion of active microelectrode sites of MS arrays observed immediately after implantation.

## 5. Conclusions

In this study, we demonstrate that there may be a depth-dependent effect on recorded single unit activity over time for both SS and MS intracortical MEAs. While numerous mechanisms may be involved in this decline of activity, our results indicate that the implantation depth of microelectrodes within the cortex resulting from particular designs needs to be considered relative to the chronic performance characterization. 

## Figures and Tables

**Table 1:** Antibody staining.

Primary Antibodies	Dilution	Secondary Antibodies	Dilution
GFAP (astrocytes)	1:500	Goat anti-chicken IgG(Alexa Fluor 647)	1:4000
NeuN (neuronal nuclei)	1:500	Goat anti-rabbit IgG (Alexa Fluor 555)	1:4000

## References

[1] Saha S., Mamun K.A., Ahmed K., Mostafa R., Naik G.R., Darvishi S., Khandoker A.H., Baumert M. (2021). Progress in Brain Computer Interface: Challenges and Opportunities. Front. Syst. Neurosci..

[2] Lebedev M.A., Nicolelis M.A. (2006). Brain-machine interfaces: Past, present and future. Trends Neurosci..

[3] Jalili R., Kanneganti A., Romero-Ortega M.I., Wallace G.G. (2017). Implantable electrodes. Curr. Opin. Electroche.

[4] Pancrazio J.J., Cogan S.F. (2019). Editorial for the Special Issue on Neural Electrodes: Design and Applications. Micromachines.

[5] Schwartz A.B., Cui X.T., Weber D.J., Moran D.W. (2006). Brain-controlled interfaces: Movement restoration with neural prosthetics. Neuron.

[6] Shoffstall A.J., Capadona J.R. (2018). Bioinspired materials and systems for neural interfacing. Curr. Opin. Biomed. Eng..

[7] van Gerven M., Farquhar J., Schaefer R., Vlek R., Geuze J., Nijholt A., Ramsey N., Haselager P., Vuurpijl L., Gielen S. (2009). The brain-computer interface cycle. J. Neural Eng..

[8] Stiller A.M., Usoro J., Frewin C.L., Danda V.R., Ecker M., Joshi-Imre A., Musselman K.C., Voit W., Modi R., Pancrazio J.J. (2018). Chronic Intracortical Recording and Electrochemical Stability of Thiol-ene/Acrylate Shape Memory Polymer Electrode Arrays. Micromachines.

[9] Deku F., Frewin C.L., Stiller A., Cohen Y., Aqeel S., Joshi-Imre A., Black B., Gardner T.J., Pancrazio J.J., Cogan S.F. (2018). Amorphous Silicon Carbide Platform for Next Generation Penetrating Neural Interface Designs. Micromachines.

[10] Guitchounts G., Cox D. (2020). 64-Channel Carbon Fiber Electrode Arrays for Chronic Electrophysiology. Sci Rep..

[11] Shin H., Jeong S., Lee J.H., Sun W., Choi N., Cho I.J. (2021). 3D high-density microelectrode array with optical stimulation and drug delivery for investigating neural circuit dynamics. Nat. Commun..

[12] Szarowski D.H., Andersen M.D., Retterer S., Spence A.J., Isaacson M., Craighead H.G., Turner J.N., Shain W. (2003). Brain responses to micro-machined silicon devices. Brain Res..

[13] Wang X.C., Hirschberg A.W., Xu H.J., Slingsby-Smith Z., Lecomte A., Scholten K., Song D., Meng E. (2020). A Parylene Neural Probe Array for Multi-Region Deep Brain Recordings. J. Microelectromech. Syst..

[14] LaPlaca M.C., Cullen D.K., McLoughlin J.J., Cargill R.S. (2005). High rate shear strain of three-dimensional neural cell cultures: A new in vitro traumatic brain injury model. J. Biomech..

[15] Pancrazio J.J., Deku F., Ghazavi A., Stiller A.M., Rihani R., Frewin C.L., Varner V.D., Gardner T.J., Cogan S.F. (2017). Thinking Small: Progress on Microscale Neurostimulation Technology. Neuromodulation.

[16] Zhu R., Huang G.L., Yoon H., Smith C.S., Varadan V.K. (2012). Biomechanical Strain Analysis at the Interface of Brain and Nanowire Electrodes on a Neural Probe. J. Nanotechnol. Eng. Med..

[17] Cogan S.F. (2008). Neural stimulation and recording electrodes. Annu. Rev. Biomed. Eng..

[18] Stiller A.M., Usoro J.O., Lawson J., Araya B., Gonzalez-Gonzalez M.A., Danda V.R., Voit W.E., Black B.J., Pancrazio J.J. (2020). Mechanically Robust, Softening Shape Memory Polymer Probes for Intracortical Recording. Micromachines.

[19] Usoro J.O., Shih E., Black B.J., Rihani R.T., Abbott J., Chakraborty B., Pancrazio J.J., Cogan S.F. (2019). Chronic stability of local field potentials from standard and modified Blackrock microelectrode arrays implanted in the rat motor cortex. Biomed. Phys. Eng. Expr..

[20] Prasad A., Sanchez J.C. (2012). Quantifying long-term microelectrode array functionality using chronic in vivo impedance testing. J. Neural Eng..

[21] Kozai T.D., Catt K., Li X., Gugel Z.V., Olafsson V.T., Vazquez A.L., Cui X.T. (2015). Mechanical failure modes of chronically implanted planar silicon-based neural probes for laminar recording. Biomaterials.

[22] Kozai T.D., Du Z., Gugel Z.V., Smith M.A., Chase S.M., Bodily L.M., Caparosa E.M., Friedlander R.M., Cui X.T. (2015). Comprehensive chronic laminar single-unit, multi-unit, and local field potential recording performance with planar single shank electrode arrays. J. Neurosci. Methods.

[23] Beaulieu C. (1993). Numerical Data on Neocortical Neurons in Adult-Rat, with Special Reference to the Gaba Population. Brain Res..

[24] DeFelipe J., Alonso-Nanclares L., Arellano J.I. (2002). Microstructure of the neocortex: Comparative aspects. J. Neurocytol..

[25] Jones E.G. (1998). Viewpoint: The core and matrix of thalamic organization. Neuroscience.

[26] Meyer H.S., Schwarz D., Wimmer V.C., Schmitt A.C., Kerr J.N., Sakmann B., Helmstaedter M. (2011). Inhibitory interneurons in a cortical column form hot zones of inhibition in layers 2 and 5A. Proc. Natl. Acad. Sci. USA.

[27] Kozai T.D., Li X., Bodily L.M., Caparosa E.M., Zenonos G.A., Carlisle D.L., Friedlander R.M., Cui X.T. (2014). Effects of caspase-1 knockout on chronic neural recording quality and longevity: Insight into cellular and molecular mechanisms of the reactive tissue response. Biomaterials.

[28] Golabchi A., Woeppel K.M., Li X., Lagenaur C.F., Cui X.T. (2020). Neuroadhesive protein coating improves the chronic performance of neuroelectronics in mouse brain. Biosens. Bioelectron..

[29] Zatonyi A., Orban G., Modi R., Marton G., Meszena D., Ulbert I., Pongracz A., Ecker M., Voit W.E., Joshi-Imre A. (2019). A softening laminar electrode for recording single unit activity from the rat hippocampus. Sci. Rep..

[30] Harris J.P., Hess A.E., Rowan S.J., Weder C., Zorman C.A., Tyler D.J., Capadona J.R. (2011). In vivo deployment of mechanically adaptive nanocomposites for intracortical microelectrodes. J. Neural Eng..

[31] Lee H.C., Ejserholm F., Gaire J., Currlin S., Schouenborg J., Wallman L., Bengtsson M., Park K., Otto K.J. (2017). Histological evaluation of flexible neural implants; flexibility limit for reducing the tissue response?. J. Neural Eng..

[32] Wen X., Wang B., Huang S., Liu T.L., Lee M.S., Chung P.S., Chow Y.T., Huang I.W., Monbouquette H.G., Maidment N.T. (2019). Flexible, multifunctional neural probe with liquid metal enabled, ultra-large tunable stiffness for deep-brain chemical sensing and agent delivery. Biosens. Bioelectron..

[33] Jiao X., Wang Y., Qing Q. (2017). Scalable Fabrication Framework of Implantable Ultrathin and Flexible Probes with Biodegradable Sacrificial Layers. Nano Lett..

[34] Potter K.A., Jorfi M., Householder K.T., Foster E.J., Weder C., Capadona J.R. (2014). Curcumin-releasing mechanically adaptive intracortical implants improve the proximal neuronal density and blood-brain barrier stability. Acta Biomater..

[35] Deku F., Cohen Y., Joshi-Imre A., Kanneganti A., Gardner T.J., Cogan S.F. (2018). Amorphous silicon carbide ultramicroelectrode arrays for neural stimulation and recording. J. Neural Eng..

[36] Kozai T.D., Catt K., Du Z., Na K., Srivannavit O., Haque R.U., Seymour J., Wise K.D., Yoon E., Cui X.T. (2016). Chronic In Vivo Evaluation of PEDOT/CNT for Stable Neural Recordings. IEEE Trans. Biomed. Eng..

[37] Chung J.E., Joo H.R., Fan J.L., Liu D.F., Barnett A.H., Chen S., Geaghan-Breiner C., Karlsson M.P., Karlsson M., Lee K.Y. (2019). High-Density, Long-Lasting, and Multi-region Electrophysiological Recordings Using Polymer Electrode Arrays. Neuron.

[38] Guan S., Wang J., Gu X., Zhao Y., Hou R., Fan H., Zou L., Gao L., Du M., Li C. (2019). Elastocapillary self-assembled neurotassels for stable neural activity recordings. Sci. Adv..

[39] Massey T.L., Santacruz S.R., Hou J.F., Pister K.S.J., Carmena J.M., Maharbiz M.M. (2019). A high-density carbon fiber neural recording array technology. J. Neural Eng..

[40] Defelipe J., Farinas I. (1992). The Pyramidal Neuron of the Cerebral-Cortex—Morphological and Chemical Characteristics of the Synaptic Inputs. Prog. Neurobiol..

[41] Kao C.Q., Goforth P.B., Ellis E.F., Satin L.S. (2004). Potentiation of GABA(A) currents after mechanical injury of cortical neurons. J. Neurotrauma.

[42] Keating C.E., Cullen D.K. (2021). Mechanosensation in traumatic brain injury. Neurobiol. Dis..

[43] Magou G.C., Pfister B.J., Berlin J.R. (2015). Effect of acute stretch injury on action potential and network activity of rat neocortical neurons in culture. Brain Res..

[44] Stiller A.M., Black B.J., Kung C., Ashok A., Cogan S.F., Varner V.D., Pancrazio J.J. (2018). A Meta-Analysis of Intracortical Device Stiffness and Its Correlation with Histological Outcomes. Micromachines.

